# Development of a Mobile Phone App to Promote Safe Sex Practice Among Youth in Stockholm, Sweden: Qualitative Study

**DOI:** 10.2196/12917

**Published:** 2020-01-28

**Authors:** Anna Nielsen, Aspasia Bågenholm, Ayesha De Costa

**Affiliations:** 1 Karolinska Institutet Department of Women's and Children's Health Stockholm Sweden; 2 Karolinska Institutet Department of Public Health Sciences Stockholm Sweden

**Keywords:** mHealth, youth, sexual health, condoms, Sweden

## Abstract

**Background:**

Mobile health (mHealth) has been shown to be effective in increasing knowledge of sexual health among youth. To date, evaluations mostly refer to interventions delivered via computer, email, and text messages. The possibility of downloading apps on mobile devices has opened up opportunities to develop engaging interventions on safe sexual health promotion. To attract young users and have them engage with a sexual health app, it is important to involve youth in intervention development.

**Objective:**

This study aimed to obtain input from youth on the content of a mobile phone app intended to promote safe sex and increase condom use among youth in Stockholm.

**Methods:**

This study was conducted at the Youth Health Clinics (YHC) in Stockholm County, Sweden. A total of 15 individual in-depth interviews and 2 focus group discussions (with youth aged 18-23 years) were conducted at the YHC in Stockholm. Areas explored were: (1) youth perceptions of condom use (advantages and obstacles), (2) perceptions of mHealth to promote safe sexual practices, and (3) content development for a mobile phone app to promote safe sex.

**Results:**

The smartphone app was developed based on the categories that emerged from the data. With regard to content, youth requested sex education, including information on sexually transmitted infections. In addition, condom-specific information, including practical usage technique, advice on how to have the condom talk, and how to decrease shame related to condom use, was requested. Youth suggested different modes to deliver the content, including text messages, movie clips, and push notifications. It was suggested that the tone of the messages delivered should be fun, entertaining, and supportive. The inputs from youth influenced the development of the following sections of the app: *Condom Obstacles and Solutions*; *Quiz*; *Games*; *Self-Refection*; *Challenges*; *Stories by Peers* (stories from peers and information from a doctor); *Condom Tips, Pep Talk, and Boosting*; and *Random Facts*.

**Conclusions:**

It is important to use input from youth when developing a smartphone intervention since the success of the intervention largely depends on the level of engagement and usage by youth. Furthermore, if proven efficient in increasing condom use, it is important that the development, including content and mode, is thoroughly described so that the intervention can be replicated. Likewise, if proven inefficient, it is important to learn from mistakes to improve and adjust the intervention. The effect of this smartphone app on safe sexual practices among youth is being evaluated in a pragmatic randomized controlled trial in Stockholm (ISRCTN13212899) and will be reported separately.

## Introduction

### Background

Sexual risk taking, including multiple sexual partners, sex outside stable relationships, and sex without a condom, has increased among Swedish youth over the past years [1-3]. In addition, *Chlamydia trachomatis* infections have steadily increased since the mid-90s, and young people aged 15 to 29 years account for 85% of all infections in the country [4]. These reports indicate that efforts, such as sex education in schools, accessibility to youth friendly clinics, and widespread testing for sexually transmitted infections (STIs), need to be complemented with new innovative interventions to reduce the burden of these infections among youth.

Mobile health (mHealth), that is, using mobile devices to address health priorities, has been shown to be effective in promoting elements of sexual health, such as increased broad knowledge regarding sexual health, increased testing for STIs, and condom use, among youth [5,6]. To date, evaluations mostly refer to interventions delivered via computer, email, and text messages [5,6]. The possibility of downloading apps on mobile devices has opened new opportunities to develop and distribute engaging health promotion interventions [7]. Health promotion via apps is suitable for youth who are a tech-savvy population that spends a significant amount of time on their mobile phones. It has been reported that this population checks their smartphones as often as 300 times per day [8]. In addition, the coverage of mobile phone ownership is high; 98% of the Swedish population owns a mobile phone, and 92% of these are smartphones [8]. To attract young users and get youth to engage with an app on sexual health, it is important to involve the target group in the development of the intervention [9-11].

We developed a mobile phone app to increase sexual health and condom use among youth. We used 2 different models of behavioral change. The transtheoretical model (TTM) is a model that conceptualizes the process of intentional behavior change and includes the following different stages of change [12]: *precontemplation—*not ready for a change, *contemplation*—getting ready for a change, *preparation*—intends to take action within the foreseeable future and has taken some behavioral steps in this direction, *action*—changed behavior, and *maintenance*—adheres to the new behavior. In a subsequent randomized controlled trial (RCT), youth with high-risk sexual behavior will be included. We, therefore, assumed users of the app to be at the *precontemplation* stage when entering the intervention study. The app was designed to help participants move through the different stages of change toward a new behavior. The second integrated behavioral model (IBM) contains 5 components affecting behavior [13]: *behavioral intention*, which is determined by attitudes, perceived norms, and personal agency; *knowledge and skills* to carry out the behavior; *importance to the individual*, *environmental constraints* that make behavioral performance difficult; and *habit* (experience performing the behavior and the behavior will become habitual). The IBM was taken into account while developing different self-reflecting exercises in the app.

### Objectives

The objective of this study was to describe exploratory work with youth from Stockholm County to obtain their input on the content of a mobile phone app to promote condom use. Feedback from participants are to be incorporated subsequently into the app, which will then be tested at the Youth Health Clinics (YHC) in Stockholm County.

## Methods

### Setting

This study was conducted at the YHC in Stockholm County, Sweden. There are 250 clinics in the whole country and 33 in Stockholm County [14,15].

### Participant Selection

A total of 15 individual in-depth interviews with youth at the YHC and 2 focus group discussions (FGDs) were conducted. Interviewed participants were selected purposefully using heterogeneous sampling [16] so that youth from different socioeconomic areas were represented. For the FGDs, youth who were clients of the YHC were invited to participate. Sociodemographic characteristics of the participants are presented in Table 1.

**Table 1:** Sociodemographic characteristics of participants in individual interviews and focus group discussions.

Population characteristics		Individual interviews (n=15)	Focus group discussions (n=10)	Total (N=25)
**Sex, n**				
	Male	7	4	11
	Female	8	6	14
Age (years), range		18-22	18-23	18-23
**Origin, n**				
	Swedish	10	9	19
	Non-Swedish	5	1	6
**Socioeconomic, n**				
	High	3	5	8
	Middle	6	3	9
	Low	6	2	8

### Data Collection

Interviews with youth aged 18 to 23 years were performed from April 2015 to August 2016 (7 males and 8 females). Interviews with youth were performed by the first author (AN) in Swedish. They were recorded and transcribed verbatim. The FGDs were held in October 2016 and November 2016. The first FGD had 4 female participants, whereas the second FGD had 2 females and 4 males. The FGDs, supported by 3 members of the research team, were recorded; notes were taken; and a summary of notes and the recordings were made. Individual interviews were conducted to enable exploration of the sensitive subject of sexual health, and FGDs were held to allow for inspiring exchanges and development of new ideas. All participants stated heterosexual preferences.

### Areas Explored

The questions posed in the interviews and FGDs originated from preknowledge regarding low condom use among youth in Sweden and the current primary and secondary prevention strategies for STIs. The areas explored were as follows:

- Why do you think condom use is low among Swedish youth?
- What is positive with condom use?
- What do you think about mobile phones to reach young people with health messages?
- Could a smartphone app work to mediate safe sexual practices?
- What would such an app contain?
- How could the app be made attractive to youth?
- Mode and timing of push notifications (message/reminders to engage in activities) in the app.

### Data Analysis

The recordings and transcripts were listened to and read through on several occasions. Data were divided into 3 different categories, which were directly related to the areas explored and the suggestions from youth regarding what the app should contain, how the content should be delivered, and what tone should be used. Subcategories that emerged from the data were organized under each of these main categories as a result of parallel analysis and mutual in-depth discussions between researchers.

### Planned Duration of the Intervention

The intervention duration was intended to be 180 days. This duration was chosen based on previous research that had shown a positive effect on condom use for an mHealth intervention lasting less than 6 months, with decreasing effect over longer follow-up periods [5].

### Ethical Considerations

Written informed consent was obtained from each participant. The study was approved by the Stockholm Regional Ethical Board (reference number 2013/1399-31/2, with amendment 2015/739-32).

## Results

The team that developed the app had varied backgrounds, including YHC staff working with youth (eg, midwife) and in public health, medicine, information technology (IT), and behavioral science. The smartphone app (subsequently named *Skyddslaget* or *Protection Team*) was developed based on the main categories and subcategories that emerged from the data from interviews and FGDs. The main categories were app content, app mode, and app tone. Suggestions for the content, mode, and tone and the subcategories are presented in Textbox 1.

Discussions regarding low condom use among the youth in Sweden and obstacles to condom use (eg, embarrassing to mention and loss of sensation) as well as positive aspects of using protection generated subcategories under app content. For example, youth requested sex education, including STI information, and, additionally, condom-specific information, including practical usage technique. Youth also requested advice on how to have *the condom talk* and decrease shame related to condom use.

While discussing how the app should be made attractive to youth, study participants suggested different modes to deliver the content, including text messages, movie clips, and push notifications. To retain the interest in the app, new information should be added into the app. Youth suggested that the tone of the messages delivered should be fun, entertaining, and supportive. In addition, youth requested that content from peers should be added.

During the subsequent process, the inputs from youth (Textbox 1) influenced the development of the following different sections of the app: *Condom Obstacles and Solutions*; *Quiz*; *Games*; *Self-Refection*; *Challenges*; *Stories by Peers* (stories from peers and information from a doctor); *Condom Tips, Pep Talk, and Boosting;* and *Random Facts.* The different sections of the app were related to the content, mode, and tone requested by participants. For example, in the *Condom Obstacles and Solutions* section, information on condom technique and exercises on how to suggest a condom to your partner were included. The names of the sections and the material presented in the different sections were developed by the research team. As described, the TTM was chosen as the theoretical framework for intervention development. In the modified TTM, each stage lasted for 30 days and aimed to help youth move from one stage to the next and thereby achieve behavioral change.

At the beginning of the intervention, the precontemplation phase, the messages included general educative information regarding the benefits of safe sexual practices, taking into account that the participants were not yet ready for a change. As the intervention evolved, the content became more oriented toward behavioral change by asking participants to reflect upon individual reasons behind sexual risk taking and benefits of a new behavior. Toward the end, the messages aimed to support the new behavior.

**Requests from youth regarding the content, mode, and tone of the app.**

**Content**

- Information regarding sexually transmitted infection
- Sex education
- Condom information
- Increase self-confidence related to condom use
- Condom technique
- Preparation for the condom talk
- Decrease shame and stigma related to condom use
- Alcohol and unsafe sex
- Questioning norms (sex with condom is not good)
- Normalizing condoms

**Mode**

- Games
- Weekend condom reminders
- Movie clip
- Imagery (emojis)
- Text (not heavy)
- Quiz
- Push notifications
- Adding new information to retain interest
- Interactive

**Tone**

- Fun and entertaining
- Supportive
- Containing pep talk
- Allowing
- Scaring (the “light version”)
- Identifying with peers
- Encouraging

**Figure 1:**
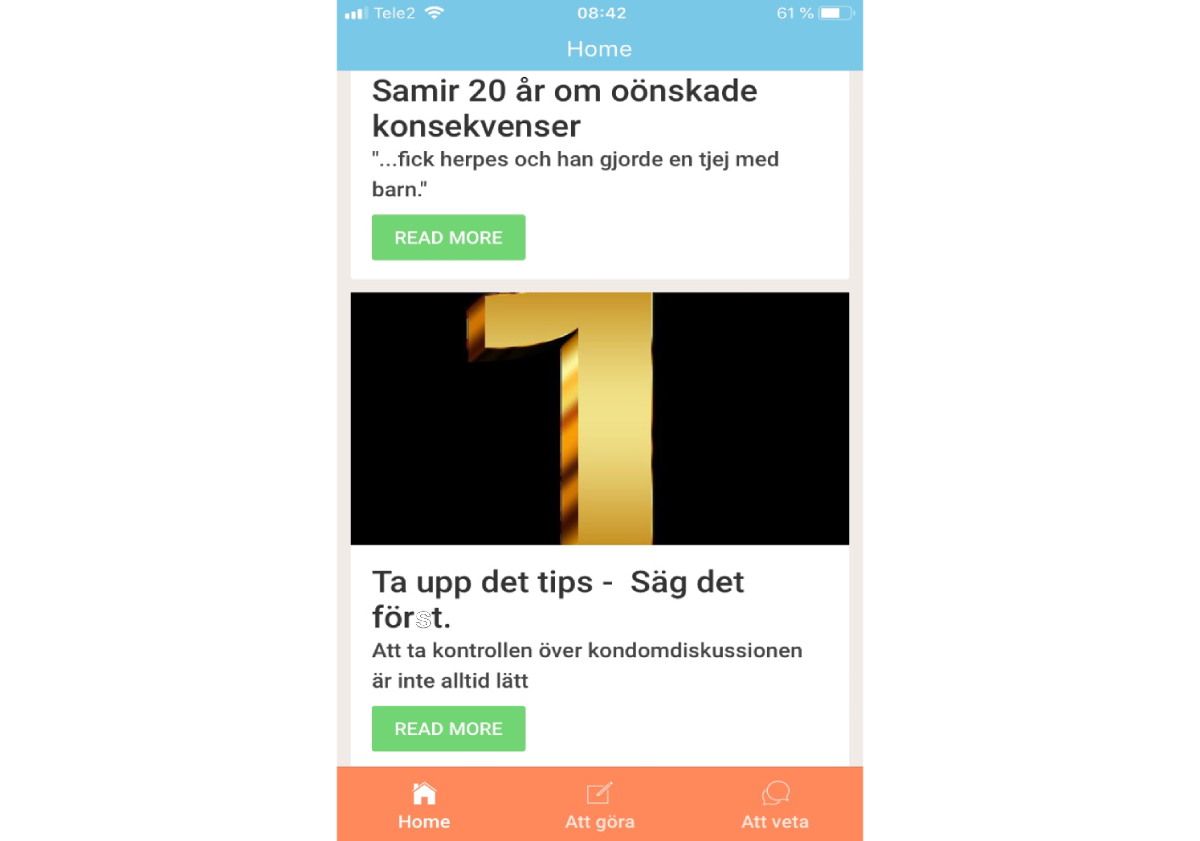
Screenshot of the intervention.

**Figure 2:**
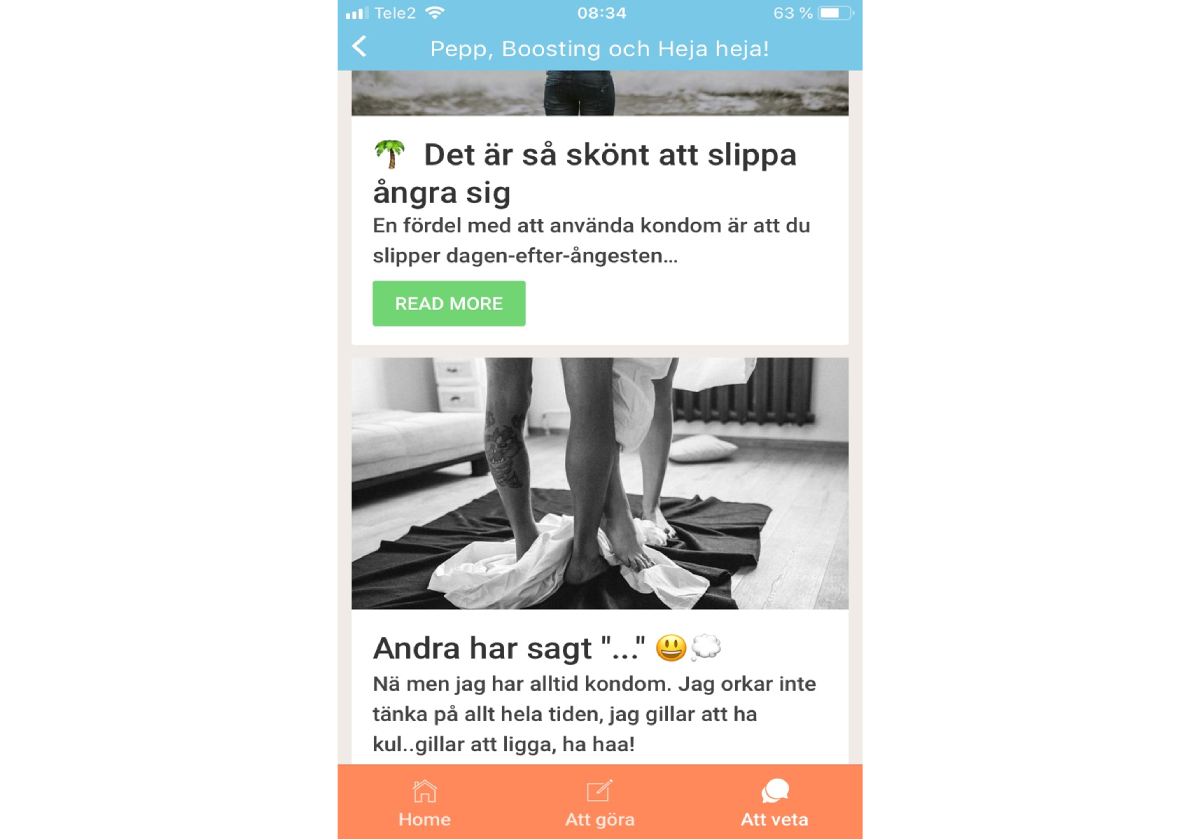
Screenshot of the intervention.

The self-reflecting and pedagogical tasks in the app were based on the IBM. These tasks addressed feelings, thoughts, experiences, attitudes, and perceived self-control in relation to condom use and unprotected sex and were mainly found under the *Condom Obstacles and Solutions* and *Self-Reflection* categories*.* Self-reflection was introduced during the preparation and action phases. Identification with peers was strongly suggested by youth and was included in the *Stories by Peers* category (peers tell their story), and this aimed to create a sense of identification and thereby eliminate possible feelings of shame related to a behavior. On each day of the intervention, at least one condom tip was posted (under the category *condom tip*). The aim was to inform about different condom types and sizes and to normalize condoms by daily exposure. In addition, push notifications were used to inform users that new content had been added into the app. Every Friday evening, a condom reminder push notification was sent out to participants. Figures 1 and 2 show details of the app (screenshots). Table 2 presents the different sections of the app with the TTM stages of change.

Content was added to the app on a daily basis for 180 days. The function and content of the app were initially piloted among 10 users. The pilot users requested more attention to be drawn to the app during the first weeks of usage; this resulted in an increase in the number of push notifications sent to the participants during the first and second week of the intervention. In addition, there were a number of minor technical support issues from the app development side that needed attention. For example, when opening the push notifications, the user was not redirected to the app, and all weekend reminders (N=26) were posted on the first Friday of the intervention.

**Table 2:** App sections with examples of content in the different stages of change according to the transtheoretical model.

App sections	Precontemplation (no intention to take action)	Contemplation (intends to take action)	Preparation (ready to act toward safer behavior)	Action/decision (changed behavior)	Action/normalize (normalizing new behavior)	Maintenance (maintaining new behavior)
Condom Obstacles and Solutions	Practice makes perfect—use a condom during self-sex	Examples of comebacks if someone refuses a condom	Finding the best fit in relation to size and shape	Let go of negative attitudes toward condoms	—^a^	Advanced comebacks related to noncondom users
Quiz	Professional condom user or beginner? Do the test here.	Would you have sex with someone who…?	Will you stick with your condom decision when drunk?	Which lubricant to which condom?	About different STIs^b^	Make condoms sexy
Games	—	—	Icebreaker: discuss condom use with friends	Discuss condoms in an intimate way with a partner	—	—
Self-Reflection	How would you feel if someone suggested a condom?	Are you affected by others’ opinions about condoms?	Are you letting your feelings take over your decision?	Holding on to your decision	Are you letting others decide what/who/when/how?	Looking back: summarizing new knowledge and behavior…
Challenges	—	—	—	Bring a condom with you for the coming 10 days	Talk about condoms, mention condoms	Pick your own challenge for the future
Stories by Peers	Doctor provides information on different STIs	Eric, 22 years: “I sometimes think about what can happen if I don’t use a condom.”	Philippe, 21 years: “She kept the baby and moved abroad.”	Kim, 20 years, on getting herpes, “It is so not worth it.”	Ellen, 18 years, on alcohol and unsafe sex	Adina, 21 years: “I took the decision early on to always use a condom.”
Condom tip	Condom of the day	Condom of the day	Condom of the day	Condom of the day	Condom of the day	Condom of the day
Pep talk	Increase the chance of using a condom by bringing one	Expose yourself to condoms	Love yourself—you do not have to agree to have sex without a condom	Do not forget to practice your new skills	Forget the feelings of embarrassment, just say it	You have all the knowledge now
Random facts	The World Health Organization’s definition of sexual health	Did you know circumference is the most important aspect of condom fit?	Which lubricant for which condom	Information about femindom	History of the condom	Anecdotes

^a^Not applicable.

^b^STI: sexually transmitted infection.

## Discussion

We used the data from FGDs and individual interviews with youth belonging to the target group to develop the content, mode, and tone of a mobile phone app to promote safe sex. Heterogeneous sampling was used, which allowed for input from youth of different backgrounds to be explored. It is important to use input from youth as the success of the intervention largely depends on their level of engagement and usage. The content and the mode of delivery were directly obtained from discussions with youth, which could possibly increase the level of interest among the target group. Input from youth were embedded into existing frameworks that were previously proven to be successful (TTM and IBM) [17].

A program delivered via a mobile phone app is unique as most previously evaluated mHealth interventions for sexual health were delivered via text messages and websites [5]. In addition, the intervention time of 180 days is longer than what was previously reported. Previous evaluations of the effects of technology-based programs indicate that, specifically for condom use, stronger effects were found in short-term interventions (ie, 1- to 5-month follow-up) compared with studies that evaluated intervention effects for a duration of 6 months or more [5,6]. The lack of long-term sustainability is a problem with the short-duration interventions previously reported. There is no report on the sustainability of effect with long-term (≥6 months) interventions.

This study presents a number of valuable lessons for future app developers. Based on the strengths and limitations of the process we used to develop our app, the following should be taken into consideration:

- Organize more FGDs so that a variety of views, some possibly divergent, can be obtained. Discussions among the project team members on divergent views better inform intervention development.
- Be present during interactions with youth to raise the right questions and address limitations of the technology. Health professionals should also be present.
- Run past iterative versions of the intervention as it is in the process of development with users, so that there are multiple opportunities to test and change. This requires an investment of time over a reasonably long period, which was a constraint during the development of the intervention in this study.
- Once the intervention has been developed, pilot test for a month with a small closed group to ensure any persisting bugs are removed.

mHealth interventions are particularly suitable for youth and sexual health promotion as the intervention is delivered in a familiar and discrete way to at-risk population [18]. Analysis of the *MObile phone for SEXual health in Youth* trial where the *Skyddslaget* app was subsequently evaluated in a pragmatic RCT will be reported separately [19].

## Abbreviations

FGD: focus group discussion
IBM: integrated behavioral model
IT: information technology
mHealth: mobile health
RCT: randomized controlled trial
STI: sexually transmitted infection
TTM: transtheoretical model
YHC: Youth Health Clinics
